# Neoantigen-specific stem cell memory-like CD4^+^ T cells mediate CD8^+^ T cell-dependent immunotherapy of MHC class II-negative solid tumors

**DOI:** 10.1038/s41590-023-01543-9

**Published:** 2023-07-03

**Authors:** Spencer E. Brightman, Angelica Becker, Rukman R. Thota, Martin S. Naradikian, Leila Chihab, Karla Soria Zavala, Ashmitaa Logandha Ramamoorthy Premlal, Ryan Q. Griswold, Joseph S. Dolina, Ezra E. W. Cohen, Aaron M. Miller, Bjoern Peters, Stephen P. Schoenberger

**Affiliations:** 1 Division of Developmental Immunology, La Jolla Institute for Immunology, La Jolla, CA USA; 2 Biomedical Sciences Program, School of Medicine, University of California San Diego, La Jolla, CA USA; 3 Division of Vaccine Discovery, La Jolla Institute for Immunology, La Jolla, CA USA; 4 Division of Hematology and Oncology, University of California San Diego Moores Cancer Center, UCSD, La Jolla, CA USA; 5 Department of Medicine, University of California San Diego, La Jolla, CA USA

**Keywords:** Immunology, Diseases

## Abstract

CD4^+^ T cells play key roles in a range of immune responses, either as direct effectors or through accessory cells, including CD8^+^ T lymphocytes. In cancer, neoantigen (NeoAg)-specific CD8^+^ T cells capable of direct tumor recognition have been extensively studied, whereas the role of NeoAg-specific CD4^+^ T cells is less well understood. We have characterized the murine CD4^+^ T cell response against a validated NeoAg (CLTC_H129>Q_) expressed by the MHC-II-deficient squamous cell carcinoma tumor model (SCC VII) at the level of single T cell receptor (TCR) clonotypes and in the setting of adoptive immunotherapy. We find that the natural CLTC_H129>Q_-specific repertoire is diverse and contains TCRs with distinct avidities as measured by tetramer-binding assays and CD4 dependence. Despite these differences, CD4^+^ T cells expressing high or moderate avidity TCRs undergo comparable in vivo proliferation to cross-presented antigen from growing tumors and drive similar levels of therapeutic immunity that is dependent on CD8^+^ T cells and CD40L signaling. Adoptive cellular therapy (ACT) with NeoAg-specific CD4^+^ T cells is most effective when TCR-engineered cells are differentiated ex vivo with IL-7 and IL-15 rather than IL-2 and this was associated with both increased expansion as well as the acquisition and stable maintenance of a T stem cell memory (T_SCM_)-like phenotype in tumor-draining lymph nodes (tdLNs). ACT with T_SCM_-like CD4^+^ T cells results in lower PD-1 expression by CD8^+^ T cells in the tumor microenvironment and an increased frequency of PD-1^+^CD8^+^ T cells in tdLNs. These findings illuminate the role of NeoAg-specific CD4^+^ T cells in mediating antitumor immunity via providing help to CD8^+^ T cells and highlight their therapeutic potential in ACT.

## Abstract (web summary)

In cancer, neoantigen (NeoAg)-specific CD8^+^ T cells capable of direct tumor recognition have been extensively studied but little is known of the role of NeoAg-specific CD4^+^ T cells. Here Schoenberger and colleagues analyze an oligoclonal CD4^+^ T cell response to a naturally arising murine tumor NeoAg at the level of TCR usage and functionality.

## Main

Neoantigen (NeoAg)-specific T cells are frequently observed in the tumor microenvironment (TME) and periphery of human patients with cancer before and during treatment with immunotherapies such as immune checkpoint blockade (ICB) and personalized cancer vaccines^1–8^. While it is known that NeoAg-specific CD8^+^ T cells are capable of directly recognizing and destroying tumor cells, clinical responses have also been observed in patients receiving adoptive cellular therapy (ACT) with autologous tumor-infiltrating lymphocytes (TILs) containing NeoAg-specific CD4^+^ T cells, suggesting that CD4^+^ T cells also play a crucial role in directing tumor immune responses^9–11^. In human melanoma, infiltration of NeoAg-specific CD4^+^ T cells is associated with antitumor effector phenotypes of macrophages, B cells and CD8^+^ T cells^12^. Several mechanisms of antitumor immunity mediated by CD4^+^ T cells have been proposed by studies in mouse models, including direct cytotoxicity dependent on recognition of MHC-II^+^ tumor cells, local secretion of effector cytokines in the TME and providing T cell help for CD8^+^ T cells^13–15^; however, how key characteristics such as TCR avidity and cellular differentiation states impact CD4^+^ T cell-mediated antitumor immunity remains unknown.

In the present study, we identified four distinct TCR clonotypes recognizing an epitope derived from a mutated clathrin heavy chain gene (CLTC_H129>Q_) in the SCC VII tumor model. We found that CLTC_H129>Q_-specific T cell receptors (TCRs) differed in their avidity for antigen but were nonetheless similarly able to undergo antigen-dependent expansion in vivo and provide CD8^+^ T cell- and CD40L-dependent protection from tumor challenge. Furthermore, treatment with interleukin (IL)-7 and IL-15 induced a durable T stem cell memory (T_SCM_)-like phenotype in TCR-engineered CLTC_H129>Q_-specific CD4^+^ T cells, enabling these cells to effectively control tumors in the therapeutic setting, highlighting the clinical relevance of TCR-engineered CD4^+^ T cells recognizing tumor-derived NeoAg.

## Results

### Expansion of CLTC_H129>Q_-specific CD4^+^ T cells correlates with protective whole-cell vaccination

**Fig. 1:**
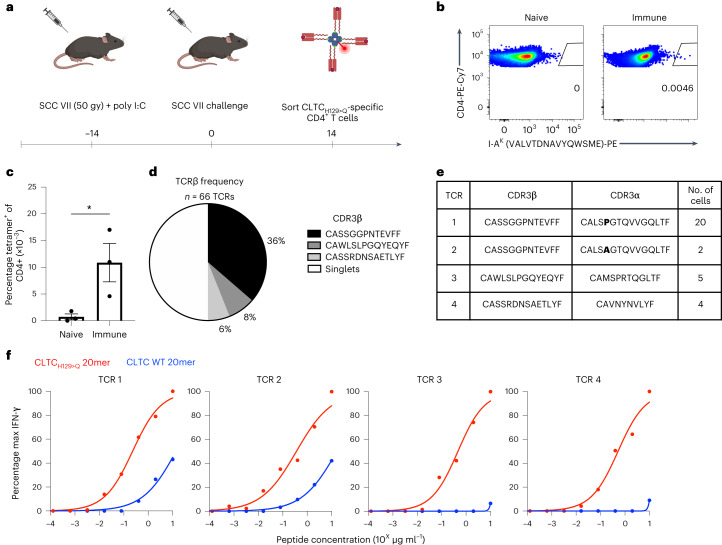
Identification and validation of CLTC_H129>Q_-specific CD4^+^ T cell clones from SCC VII. **a**, Schematic overview of tetramer sorting experiments to isolate CLTC_H129>Q_-specific T cells. **b**, Representative flow cytometry plots of tetramer-binding CD4^+^ T cells from naive or SCC VII-immune mice. **c**, Quantification of tetramer-positive CD4^+^ T cells from the spleens of naive or immunized mice; mean ± s.e.m. of three individual mice; *P = 0.049 two-tailed unpaired *t-*test. **d**, TCR β-chain diversity of tetramer-sorted T cells. **e**, CDR3 sequences for α- and β-chains of clonally expanded T cells. **f**, Concentration–response curves of IFN-γ production by primary CD4^+^ T cells retrovirally transduced with each CLTC_H129>Q_-specific TCR stimulated with splenocytes pulsed with either mutant or wild-type peptides. Data are representative of three independent experiments. Source data

**Extended Data Fig. 1:**
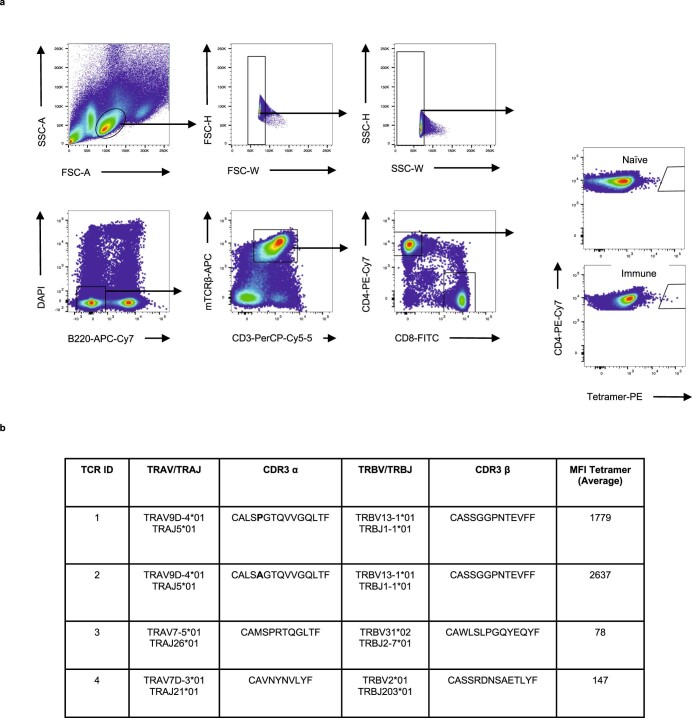
Gating strategy for sorting CLTC_H129Q_-specific CD4^+^ T cells and TCR characteristics. **a** Representative gating strategy for sorting CLTC_H129>Q_-specific CD4^+^ T cells. **b** Table of TCR genes and tetramer median fluorescence intensity for expanded T cell clones.

We have previously demonstrated that vaccination with irradiated SCC VII cells and adjuvant polyI:C is protective against subsequent live tumor challenge. SCC VII-immune mice generate CD4^+^ and CD8^+^ NeoAg-specific T cell responses, which includes recognition of a mutated clathrin heavy chain epitope (CLTC_H129>Q_) by CD4^+^ T cells^16^. To identify TCRs from CLTC_H129>Q_-specific CD4^+^ T cells, C3H/HeJ mice were immunized with irradiated SCC VII tumor cells admixed with polyI:C and challenged 14 d later with live SCC VII cells. At 14 d after tumor challenge, splenocytes from immune mice were isolated and stained with a CLTC_H129>Q_/I-A^K^ tetramer (Fig. 1a and Extended Data Fig. 1a). Consistent with our previous ELISpot results, tetramer-positive CD4^+^ T cells were present at a significantly increased frequency in SCC VII-immune mice compared to naive mice (Fig. 1b,c).

### Identification of CLTC_H129>Q_-specific CD4^+^ T cell clonotypes

To isolate CLTC_H129>Q_-specific CD4^+^ T cells, single tetramer-positive cells from challenged and protected mice were sorted into 96-well plates and the CDR3 regions of both TCR α/β-chains were amplified by PCR as previously described^17^. Sequencing of complementary DNA libraries revealed three expanded TCR β clonotypes represented among tetramer-sorted cells (Fig. 1d). These three TCR β-chains paired with four distinct α-chains, corresponding to four unique expanded T cell clones (Fig. 1e). Of note, TCR 1 and TCR 2 shared the same TCR β-chain and nearly identical α-chains, which differ at a single alanine to proline substitution within the CDR region. To confirm TCR surface assembly and specificity, each α/β receptor was expressed via retroviral transduction of naive primary C3H CD4^+^ T cells and tested for recognition of wild-type versus H129 > Q forms of the CLTC_119–133_ peptide. CD4^+^ T cells expressing each of the four TCRs produced interferon (IFN)-γ when stimulated with splenocytes pulsed with the H129 > Q peptide (Fig. 1f) but produced less or no detectable IFN-γ in response to the wild-type epitope.

### CLTC_H129>Q_-specific TCRs differ in avidity

**Fig. 2:**
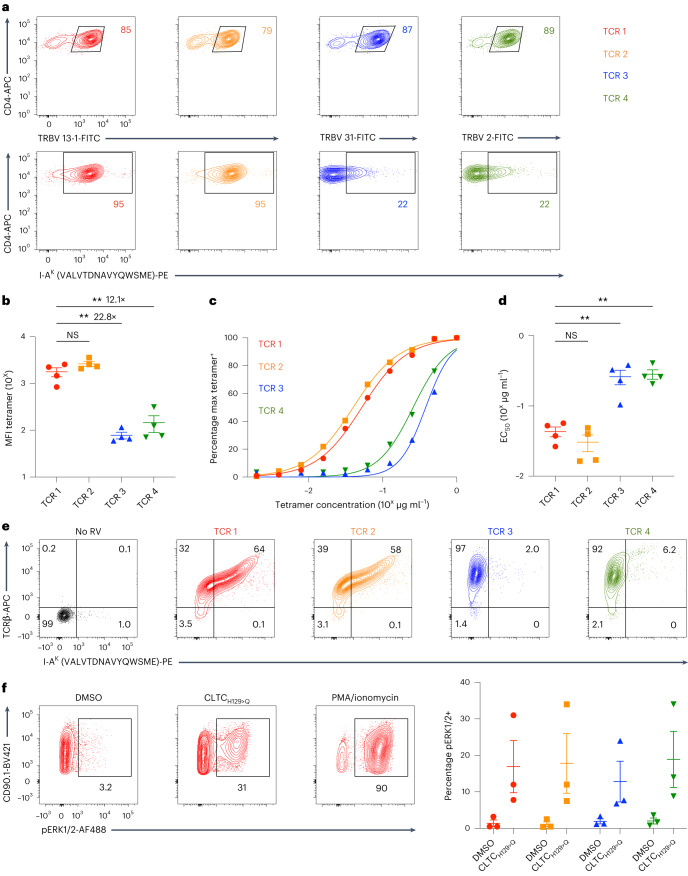
CLTC_H129>Q_-specific TCR avidity, co-receptor dependence and proximal signaling. **a**, Flow cytometry plots demonstrating expression of the introduced TCRs compared to mock transduced control T cells (top) and tetramer binding (bottom). **b**, MFI of 1 µg ml^−1^ tetramer binding to primary T cells expressing the indicated TCRs. Data represent four independent experiments, **P = 0.0024 TCR 1 versus TCR 3, *P* = 0.0033 TCR 1 versus TCR 4 one-way analysis of variance (ANOVA) with Tukey correction for multiple comparisons. NS, not significant. **c**, Tetramer titration plot demonstrating percentage of tetramer-binding cells at indicated tetramer concentrations. Data are representative of four independent experiments. **d**, EC_50_ values determined from tetramer titration curves in **c**. Data represent four independent experiments, **P = 0.0070 TCR 1 versus TCR 3, 0.0034 TCR 1 versus TCR 4 one-way ANOVA with Tukey correction for multiple comparisons. **e**, Flow cytometry plots demonstrating TCR expression and tetramer binding of CD8^+^CD4^−^58α^−^β^−^ cells expressing each TCR. Data are representative of three independent experiments. **f**, Representative flow cytometry plots of pERK1/2 staining of T cells expressing TCR 1 stimulated with naive splenocytes pulsed with dimethylsulfoxide (DMSO) or 1 µg ml^−1^ CLTC_H129>Q_ peptide (left). Quantification of pERK1/2 staining for each TCR 5 min after stimulation with naive splenocytes pulsed with DMSO or 1 µg ml^−1^ CLTC_H129>Q_ peptide. Data represent three independent experiments. All data represent mean ± s.e.m. Source data

Next, we set out to compare the functional characteristics of the CLTC_H129>Q_-specific TCRs. For all four populations of transduced primary CD4^+^ T cells, >79% of cells expressed the introduced TCR as evidenced by staining for the associated TCR β-chain variable regions (TRBVs) (Fig. 2a). Gating on the TRBV-expressing cells revealed a consistent difference in tetramer binding, with both a greater frequency and magnitude of tetramer binding observed for cells expressing TCRs 1 and 2 than those expressing TCRs 3 and 4 (Fig. 2a). Indeed, the median fluorescence intensity (MFI) of tetramer binding for cells expressing TCR 1 was significantly greater than that of cells expressing either TCR 3 or TCR 4 (22.8× and 12.1× greater, respectively) (Fig. 2b and Extended Data Fig. 1b). To further investigate the avidity differences between TCRs, we incubated transduced primary T cells with a titration of tetramer concentrations and measured the percent of maximal fluorescence at each concentration (Fig. 2c). These experiments were consistent with our initial observations, indicating that both TCRs 1 and 2 had significantly lower tetramer half-maximum effective concentration (EC_50_) values than TCRs 3 and 4 (Fig. 2d). To study the TCR-binding properties in the absence of CD4, we transduced the TCR-deficient CD8^+^ T cell hybridoma line 58α^−^β^−^ with each TCR. The 58α^−^β^−^ cells expressing TCRs 1 and 2 were able to bind tetramer independently of the CD4 co-receptor, whereas cells expressing TCRs 3 and 4 did not, despite comparable levels of TCR expression (Fig. 2e). In conclusion, the CLTC_H129>Q_-specific CD4^+^ T cell pool contains T cell clones expressing both high and moderate avidity antigen receptors.

### Differences in TCR avidity do not correlate with differences in proximal TCR signaling in vitro

Given the differences in TCR avidity observed, we investigated whether these correlate with proximal TCR signaling, as has been demonstrated in studies of tumor-specific CD8^+^ T cells^18^. To measure levels of TCR signaling, we stained permeabilized TCR-expressing primary CD4^+^ T cells for phosphorylated ERK1/2 (pERK1/2) after a brief stimulation period with peptide-pulsed splenocytes. While all four groups of TCR-expressing CD4^+^ T cells expressed higher levels of pERK1/2 after stimulation with peptide-pulsed splenocytes compared to splenocytes without added peptide, there were no significant differences in the percentage of activated cells between different TCRs (Fig. 2f). These results suggest that differences in CLTC_H129>Q_-specific TCR avidity do not correlate with differences in proximal TCR signaling.

### Expansion and activation of CLTC_H129>Q_-specific CD4^+^ T cells in vivo is TCR avidity independent

**Fig. 3:**
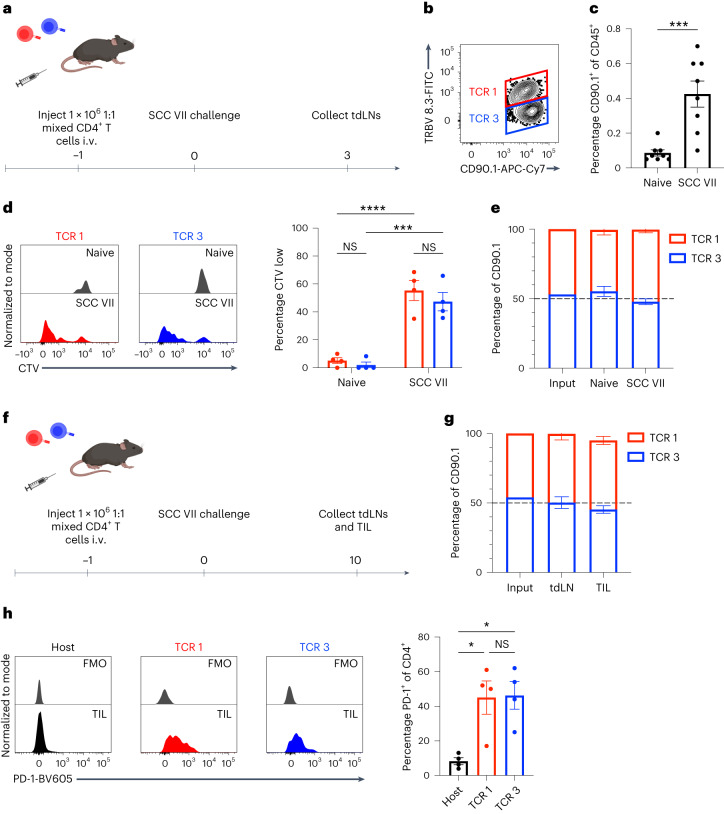
Expansion and activation of CLTC_H129>Q_-specific CD4^+^ T cells in the draining lymph node and tumor microenvironment is independent of TCR avidity. **a**, Schematic overview of co-transfer experiments performed for **c**–**e**. **b**, Representative flow cytometry plot demonstrating gating strategy to identify TCR 1- and TCR 3-expressing CD4^+^ T cells in vivo. **c**, Quantification of total CD90.1^+^ adoptively transferred CD4^+^ T cells in draining lymph nodes of naive mice or mice challenged with SCC VII tumors; *n* = 8 mice per group; ***P = 0.0006 two-tailed unpaired *t*-test. Data represent two independent experiments. **d**, Flow cytometry histograms (left) and quantification (right) of CTV dilution by TCR 1- and TCR 3-expressing CD4^+^ T cells in vivo in either naive mice or mice challenged with SCC VII tumors; *n* = 4 mice per group; ***P = 0.0003; ****P = 0.00009; two-way ANOVA with Šidák correction for multiple comparisons. Data are representative of two independent experiments. **e**, Relative frequencies of TCR 1- and TCR 3-expressing CD4^+^ T cells in the draining lymph nodes of naive and tumor-bearing mice compared to input; *n* = 4 mice per group. **f**, Schematic overview of co-transfer experiments performed for **g**,**h**. **g**, Relative frequencies of TCR 1- and TCR 3-expressing CD4^+^ T cells in the draining lymph nodes and TILs of tumor-bearing mice compared to input. *n* = 4 mice per group. **h**, Flow cytometry histograms (left) and quantification (right) of PD-1 expression by host CD4^+^ T cells compared to T cells expressing either TCR 1 or TCR 3; *n* = 4 mice per group; *P = 0.0153 host versus TCR 1; *P* = 0.0128 host versus TCR 3; one-way ANOVA with Tukey correction for multiple comparisons. Data are representative of two independent experiments. All data are represented as mean ± s.e.m. FMO, Fluorescence Minus One Control. Source data

**Extended Data Fig. 2:**
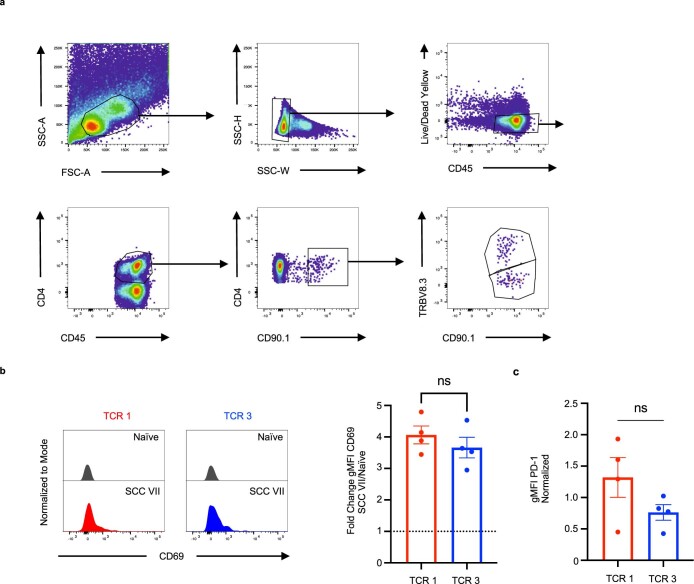
CLTC_H129Q_-specific CD4^+^ T cell activation in the tdLN and tumor during tumorigenesis. **a** Representative gating strategy to identify adoptively transferred cells expressing TCR 1 and TCR 3. **b** Representative histograms (left) and quantification (right) of CD69 upregulation by cells expressing TCR 1 and TCR 3 in the tdLN in SCC VII tumor-bearing mice compared to mice without tumors. Mean +/− SEM, *n* = 4 mice per group, representative of two independent experiments. ns p > 0.05 two-tailed unpaired T-test. **c** Quantification of PD-1 gMFI normalized to FMO for TCR 1 and TCR 3 in the tumor. Mean +/− SEM, *n* = 4 mice per group, representative of two independent experiments. ns p > 0.05 two-tailed unpaired T-test. Source data

To investigate how differences in TCR avidity may impact antigen-specific responses in vivo, we transferred equal numbers of CellTrace Violet (CTV)-labeled CD4^+^ T cells expressing either TCR 1 or TCR 3 into either naive mice or mice that were subsequently challenged with SCC VII. In this experimental system we were able to differentiate between cells expressing TCR 1 or TCR 3 as either CD90.1^+^TRBV8.3^+^ or CD90.1^+^TRBV8.3^−^, respectively (Fig. 3a,b). Antigen-specific expansion of adoptively transferred cells was apparent within 3 d, as the frequency of total CD90.1^+^ cells in tumor-draining lymph nodes (tdLNs) increased significantly in mice challenged with live SCC VII cells compared to naive animals (Fig. 3c). We found that cells expressing TCR 1 or TCR 3 proliferated to a similar extent in the context of antigen derived from live tumor cells, with no significant differences in the number of expanded CTV^low^ cells in mice challenged with SCC VII (Fig. 3d). Neither CD4^+^ T cell population proliferated significantly in naive mice, suggesting that TCR 1 does not recognize the wild-type CLTC epitope in vivo despite producing IFN-γ in response to splenocytes pulsed with high concentrations of the corresponding peptide (Figs. 3d and 1f). Consistent with these results, the relative frequency of cells expressing either TCR 1 or TCR 3 did not change significantly in either naive mice or mice challenged with SCC VII compared to their starting frequencies (Fig. 3e). In addition, both TCR 1- and TCR 3-expressing cells upregulated similar levels of the acute activation marker CD69 in an antigen-specific manner (Extended Data Fig. 2a,b). Altogether, these results suggest that NeoAg-specific CD4^+^ T cells with distinct TCRs behave similarly in vivo independent of TCR avidity.

Next, we investigated the impact of TCR avidity on T cell activation in the TME. tdLNs and tumors were collected from mice 10 d after SCC VII challenge to assess the relative frequency and activation phenotype of cells expressing either TCR 1 or TCR 3 (Fig. 3f). Consistent with our results with cells collected after 3 d, there was no significant difference in the relative frequencies of cells expressing each TCR in the tdLN or TME after 10 d (Fig. 3g). Expression of co-inhibitory markers such as PD-1 is a hallmark of both tumor-reactive CD4^+^ and CD8^+^ T cells in the TME. We determined that cells expressing either TCR 1 or TCR 3 expressed similar levels of PD-1 in the TME and both expressed significantly more PD-1 than CD90.1^−^ host CD4^+^ T cells (Fig. 3h and Extended Data Fig. 2c). These results suggest that the observed differences in TCR avidity do not influence CD4^+^ T cell persistence or activation in the TME. Given that PD-1 upregulation occurs downstream of TCR signaling, these results also suggest that CLTC_H129>Q_-specific CD4^+^ T cells are recognizing antigen in the TME as well as the tdLNs.

### CLTC_H129>Q_-specific CD4^+^ T cell protect from live tumor challenge in a CD40L and CD8^+^ T cell-dependent manner

**Fig. 4:**
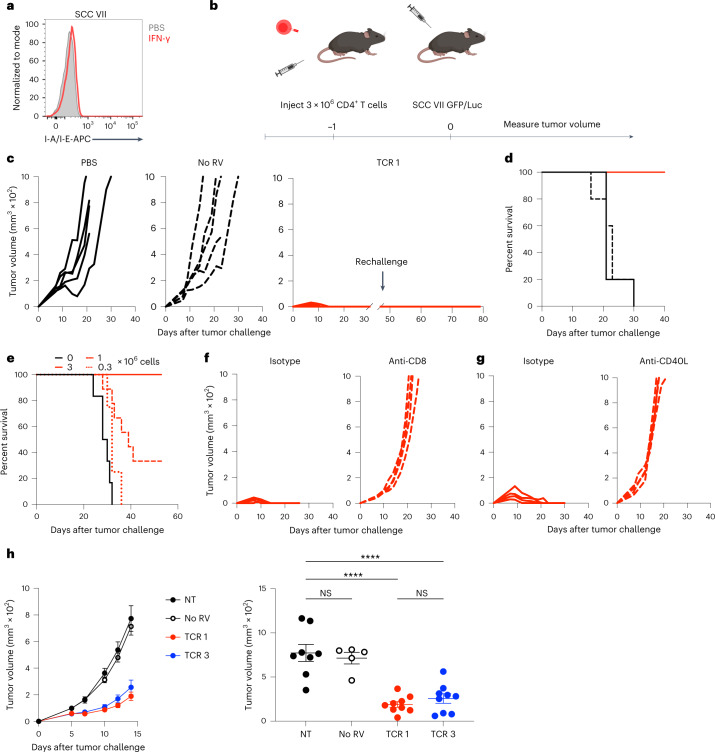
CLTC_H129>Q_-specific CD4^+^ T cells limit tumor burden in a CD8^+^ T cell- and CD40L-dependent mechanism. **a**, Flow cytometry plots indicating surface expression of I-A^K^/I-E^K^ molecules on SCC VII tumor cells in vitro after 48 h of culture in media supplemented with either phosphate-buffered saline (PBS) or 10 ng ml^−1^ IFN-γ. Data are representative of three independent experiments. **b**, Schematic of tumor protection experiments. **c**, Tumor growth curves for mice receiving PBS, 3 × 10^6^ polyclonal mock transduced T cells or 3 × 10^6^ TCR 1-expressing T cells intravenously (i.v.) 1 d before SCC VII-GFP/Luc tumor challenge. Data are representative of three independent experiments; *n* = 5 mice per group. **d**, Survival of mice shown in **c**. **e**, Survival of mice receiving the indicated number of T cells expressing TCR 1 before tumor challenge; *n* = 4 mice for 0.33 × 10^6^; *n* = 9 for 1 × 10^6^; *n* = 8 for 3 × 10^6^ and *n* = 6 for not treated (NT). Data represent two independent experiments. **f**, Tumor growth curves for mice receiving 3 × 10^6^ TCR 1-expressing T cells 1 d before SCC VII-GFP/Luc tumor challenge with concurrent injections of either anti-CD8 or isotype control antibodies; *n* = 4 mice for anti-CD8, *n* = 5 mice for isotype. Data are representative of two independent experiments. **g**, Tumor growth curves for mice receiving 3 × 10^6^ TCR 1-expressing T cells 1 d before SCC VII-GFP/Luc tumor challenge with concurrent injections of either anti-CD40L or isotype control antibodies; *n* = 4 mice per group. Data are representative of two independent experiments. **h**, Tumor growth curves for mice receiving PBS, 3 × 10^6^ polyclonal mock transduced T cells or 3 × 10^6^ TCR 1- or TCR 3-expressing T cells i.v. 1 d before SCC VII tumor challenge (left). Tumor volumes at 14 d (right); *n* = 8 mice for untreated, *n* = 5 mice receiving no retrovirus (RV) mock transduced cells and *n* = 9 mice receiving cells expressing either TCR 1 or TCR 3. Data are representative of two independent experiments. ****P = 0.00003 for NT versus TCR 1; *P* = 0.00006 for NT versus TCR 3 two-way ANOVA with Šidák correction for multiple comparisons. Data are representative of two independent experiments. Data represent mean ± s.e.m. Source data

We sought to determine whether activated CLTC_H129>Q_-specific CD4^+^ T cells could protect from live tumor challenge. Notably, SCC VII tumor cells do not express MHC-II and in vitro treatment with IFN-γ did not induce MHC-II expression, suggesting that CLTC_H129>Q_-specific CD4^+^ T cells are unable to directly recognize tumor cells even under inflammatory conditions in vivo (Fig. 4a). Notably, transfer of 3 × 10^6^ activated CD4^+^ T cells expressing the high avidity TCR 1 at 1 d before tumor challenge with SCC VII and green fluorescent protein (GFP) or luciferase (Luc) was sufficient to induce tumor rejection (Fig. 4b–d). This protection was antigen-specific, as mice receiving 3 × 10^6^ activated polyclonal CD4^+^ T cells not expressing the CLTC_H129>Q_-specific TCR had tumor growth comparable to untreated mice. None of the five protected mice receiving a subsequent tumor challenge 30 d after initial rejection developed palpable tumors, suggesting the establishment of durable immune memory. Protection by CLTC_H129>Q_-specific CD4^+^ T cells was also dependent on the frequency of these cells at the time of tumor challenge, as mice receiving either 1 × 10^6^ or 3.3 × 10^5^ cells before challenge had decreased rates of survival after inoculation of SCC VII-GFP/Luc cells (Fig. 4e).

**Extended Data Fig. 3:**
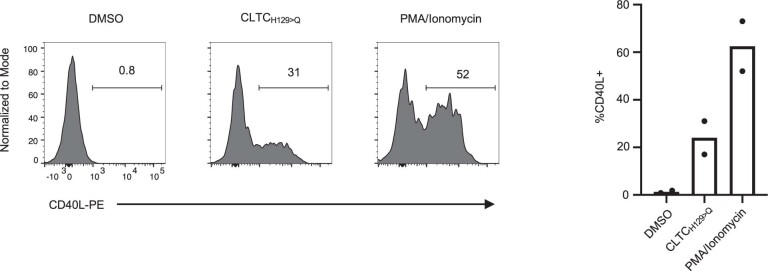
CD4^+^ T cells expressing TCR 1 upregulate CD40L upon antigen recognition. Flow cytometry histograms (left) and quantification (right) of CD40L expression by CD4^+^ T cells expressing TCR 1 stimulated with unpulsed splenocytes, splenocytes pulsed with 1 µg/mL CLTC_H129>Q_ peptide, or PMA/Ionomycin. Mean +/− SEM, Data represents two independent experiments. Source data

Given that SCC VII tumor cells do not express MHC-II (Fig. 4a), we reasoned that CLTC_H129>Q_-specific CD4^+^ T cell-mediated protection may be dependent on their role as helper cells for CD8^+^ T cells^19^. Consistent with this, depletion of CD8^+^ T cells before the transfer of CD4^+^ T cells and tumor inoculation prevented tumor rejection (Fig. 4f). Recent work has demonstrated that CD4^+^ T cells provide help to CD8^+^ T cells by CD40L-dependent licensing of cDC1s during the primary tumor response^20^. Upon stimulation with CLTC_H129>Q_ peptide-pulsed splenocytes in vitro, CD4^+^ T cells expressing TCR 1 upregulated CD40L, suggesting that this mechanism may be responsible for protection from tumor challenge (Extended Data Fig. 3). To interrogate the role of this pathway in our model, we administered anti-CD40L-blocking antibodies on the day of tumor implantation and 2 d later. Mice receiving 3 × 10^6^ activated CLTC_H129>Q_-specific CD4^+^ T cells along with anti-CD40L were no longer protected from tumor challenge (Fig. 4g). Together, these data demonstrate that NeoAg-specific CD4^+^ T cells mediate antitumor immunity by helping endogenous CD8^+^ T cells via CD40L signaling.

These findings prompted us to further probe the efficacy of CD4^+^ T cells expressing either TCR 1 or TCR 3. As antitumor efficacy was dependent on CD40L help for CD8^+^ T cells, we used the SCC VII parental cell line for all future experiments to rule out the contribution of CD8^+^ T cells specific for epitopes contained within the GFP or Luc reporter proteins. While transfer of 3 × 10^6^ cells expressing TCR 1 was no longer sufficient for complete tumor rejection against the less-immunogenic parental cell line, tumor growth was still significantly delayed compared to untreated mice or mice receiving 3 × 10^6^ polyclonal activated CD4^+^ T cells (Fig. 4h). There was no significant difference in the rate of tumor growth for mice receiving CD4^+^ T cells expressing TCR 1 or TCR 3, however, suggesting that clones with differences in TCR avidity can mediate similar CD40L- and CD8-dependent antitumor functions in vivo.

### TCR-engineered CLTC_H129>Q_-specific CD4^+^ T cells mediate therapeutic immunity

**Fig. 5:**
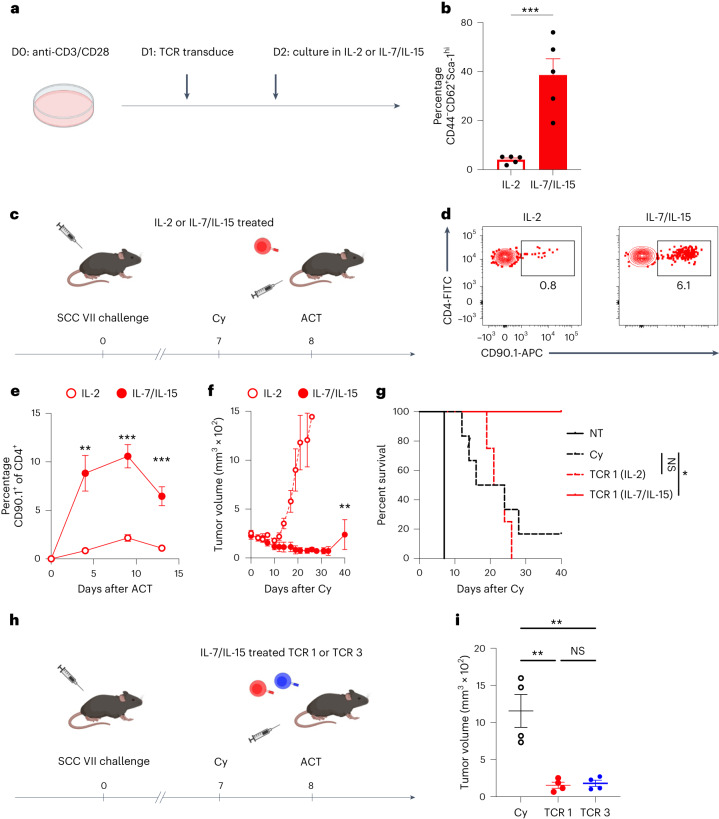
Therapeutic ACT with T_SCM_-like CLTC_H129>Q_-specific CD4^+^ T cells promotes tumor control. **a**, Schematic of culture conditions to generate TCR-engineered T_SCM_-like cells. **b**, Frequency of CD44^−^CD62L^+^Sca-1^hi^CD4^+^ T cells after culture with indicated cytokines. ***P = 0.0009; two-tailed unpaired *t*-test. Data represent five independent experiments. **c**, Schematic of therapeutic ACT experiments. **d**, Representative flow cytometry plots depicting frequency of CD90.1^+^ adoptively transferred cells in the peripheral blood of mice 4 d after adoptive transfer. **e**, Quantification of CD90.1^+^ adoptively transferred cells in the peripheral blood of mice over time after adoptive transfer. Data show mean ± s.e.m., *n* = 4 mice per group, and are representative of two independent experiments. **P = 0.0051; ***P = 0.0002 for day 9 and *P* = 0.0003 for day 13; two-tailed unpaired *t*-test. **f**, Tumor growth curves of mice treated with cyclophosphamide and 3 × 10^6^ TCR 1-expressing cells from indicated treatment groups; *n* = 4 mice per group; **P < 0.0077; two-tailed unpaired *t*-test at day 21. **g**, Survival of mice in **d**. NS, *P* = 0.74; *P = 0.025; survival *P* values by log-rank test. **h**, Schematic of therapeutic ACT with TCR 1 or TCR 3-expressing cells. **i**, Tumor volumes of mice with indicated treatments 12 d after initiation of therapy; *n* = 4 mice per group; NS, *P* = 0.99; **P = 0.0022 Cy versus TCR 1; *P* = 0.0025 Cy versus TCR 3; two-way ANOVA with Šidák correction for multiple comparisons. All data represent mean ± s.e.m. Source data

**Extended Data Fig. 4:**
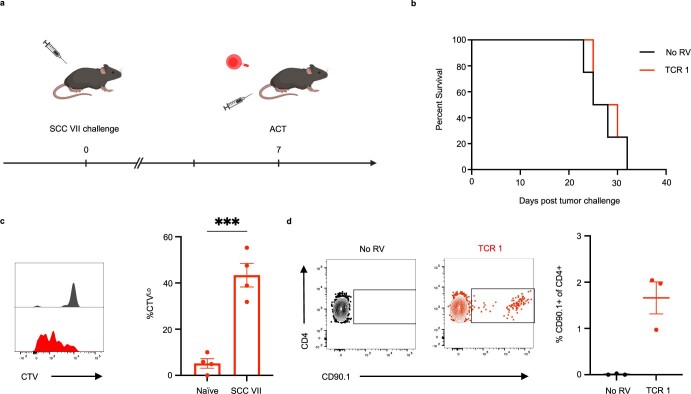
Adoptive transfer of CLTC_H129Q_-specific CD4^+^ T cells without cyclophosphamide does not improve survival. **a** Experimental scheme of therapeutic adoptive transfer experiments. **b** Survival curves comparing mice receiving 3 × 10^6^ CLTC^H129Q^-specific cells expressing TCR 1 or an equal number of mock transduced polyclonal activated CD4^+^ T cells. *n* = 4 mice per group, representative of three independent experiments. **c** Representative flow cytometry histograms (left) and quantification (right) of CellTrace violet dilution in the tdLN following adoptive transfer of CLTC^H129Q^-specific CD4^+^ T cells to either naïve mice or mice with established SCC VII tumors. Mean +/− SEM, *n* = 4 mice per group, *** p <=0.0003 two-tailed unpaired T-test. **d** Representative flow cytometry plots (left) and quantification (right) of the frequency of adoptively transferred CD90.1^+^ CD4^+^ T cells present in tumors from treated mice. Mean +/− SEM, *n* = 3 mice per group, representative of three independent experiments. Source data

**Extended Data Fig. 5:**
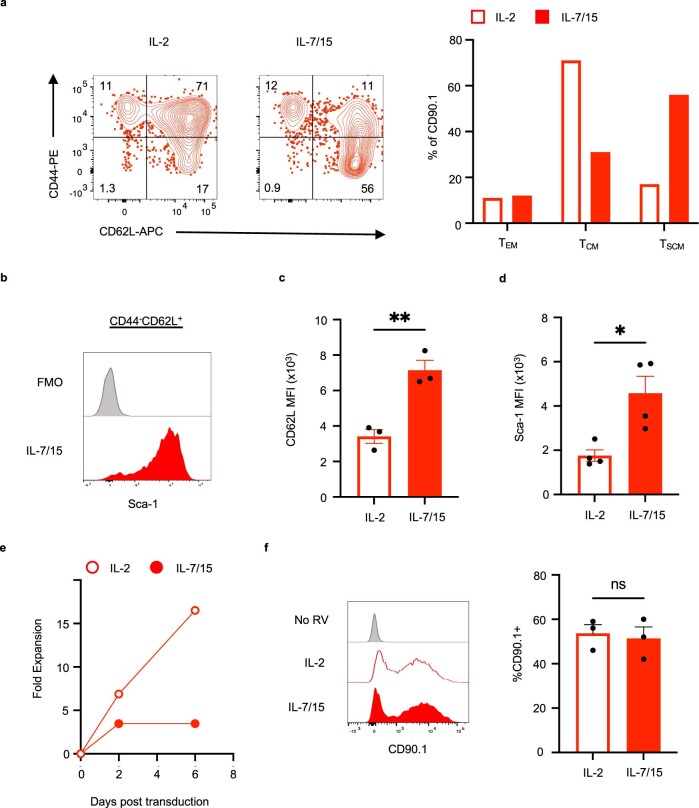
*In vitro* phenotype of TCR-engineered cells cultured in either IL-2 or IL-7 and IL-15. **a** Flow cytometry plots (left) and quantification (right) of CD44 and CD62L expression by CD4^+^ T cells cultured with indicated cytokines. T_EM_, T_CM_ and T_SCM_ are designated as CD44 + CD62L−, CD44 + CD62L + and CD44loCD62L+ respectively. Representative of four independent experiments. **b** Flow cytometry histograms of Sca-1 expression by IL-7/15-treated cells. Representative of four independent experiments. **c** Quantification of CD62L expression represented as the median fluorescence intensity for cells cultured in either IL-2 or IL-7/15. Mean +/− SEM, data represents three independent experiments. ** p = 0.0053 two-tailed unpaired T-test. **d** Quantification of Sca-1 expression represented as the median fluorescence intensity for cells cultured in either IL-2 or IL-7/15. Mean +/− SEM, data represents four independent experiments. * p = 0.0131 two-tailed unpaired T-test. **e** Fold expansion *in vitro* plotted over time for TCR-engineered cells cultured in IL-2 or IL-7/15. Data representative of four independent experiments. **f** Flow cytometry histograms (left) and quantification (right) of CD90.1 expression by transduced T cells cultured in indicated cytokines. Mean +/− SEM, data represents three independent experiments, ns p > 0.05 two-tailed unpaired T-test. Source data

Given the observation that CLTC_H129>Q_-specific CD4^+^ T cells could contribute to primary tumor immunity, we investigated the potential of TCR-engineered T cells to limit tumor burden in the context of therapeutic ACT against large established tumors. In preliminary experiments, we found that ACT with CD4^+^ T cells expressing TCR 1 did not significantly improve survival after tumor challenge compared to mice receiving non-transduced T cells, despite evidence that adoptively transferred cells were able to proliferate in the tdLNs and infiltrate tumors (Extended Data Fig. 4). We therefore sought to improve the function and survival of the engineered cells. Studies suggest that CD8^+^ T cells primed under conditions that preferentially generate less-differentiated stem cell memory T (T_SCM_) cells have a greater capacity for persistence and tumor destruction when adoptively transferred into tumor-bearing animals^21^. The common γ-chain cytokines IL-7 and IL-15 have been demonstrated to preferentially generate and expand T_SCM_-like cells from ex vivo-stimulated, naive human CD8^+^ T cells^22^. We therefore sought to determine whether culturing our TCR-engineered CD4^+^ T cells in IL-7/IL-15, rather than IL-2, could generate T_SCM_-like cells for ACT (Fig. 5a). CD4^+^ T cells cultured in IL-7/IL-15 after TCR transduction demonstrated a significant increase in the frequency of CD44^−^CD62^+^Sca-1^hi^ cells, consistent with a T_SCM_-like phenotype (Fig. 5b and Extended Data Fig. 5a–d). Cells cultured in IL-7/IL-15 were also less proliferative in vitro compared to cells cultured in IL-2 (Extended Data Fig. 5e). Despite these differences in in vitro phenotype, the TCR transduction efficiency was comparable between both treatment groups as determined by CD90.1 expression (Extended Data Fig. 5f).

To compare the in vivo expansion and therapeutic efficacy of IL-7/IL-15-treated T_SCM_-like cells with those cultured in IL-2, we transferred equal numbers (3 × 10^6^) of TCR-engineered cells from either culture condition into mice with established SCC VII tumors 1 d following treatment with the lymphodepleting chemotherapy agent cyclophosphamide (Cy) (Fig. 5c). Notably, as soon as 4 d after transfer there was a roughly tenfold increase in the frequency of CD90.1^+^ cells in the peripheral blood of mice receiving the IL-7/IL-15-treated cells, with this population peaking in size 9 d following adoptive transfer (Fig. 5d,e). Furthermore, animals receiving the T_SCM_-like CD4^+^ T cells had significantly delayed tumor growth compared to mice receiving the same number of T cells expanded in IL-2 (Fig. 5f). ACT with CD4^+^ T cells cultured in IL-7/IL-15, but not IL-2, provided a significant increase in survival beyond the direct effects of the Cy chemotherapy (Fig. 5g). Consistent with our previous results, therapeutic ACT with T_SCM_-like CD4^+^ T cells expressing TCR 3 delayed tumor growth similarly to ACT with cells expressing TCR 1 (Fig. 5h,i). These results suggest that TCR-engineered NeoAg-specific CD4^+^ T cells differentiated in IL-7/IL-15 can be effective as a therapeutic ACT treatment.

### T_SCM_-like CLTC_H129>Q_-specific CD4^+^ T cells are maintained in the tdLNs

**Fig. 6:**
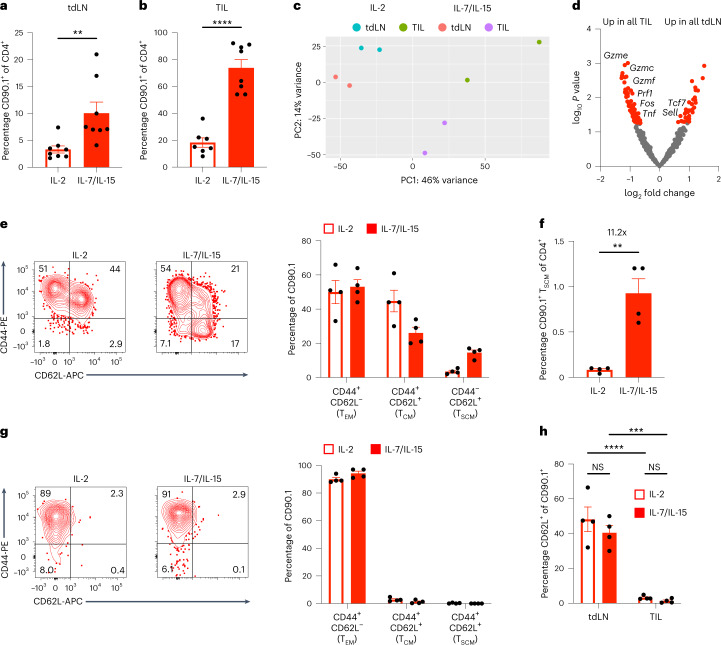
CD4^+^ T_SCM_-like cells are selectively maintained in the tumor-draining lymph nodes. **a**, Quantification of CD90.1^+^ adoptively transferred cells in the tdLNs of mice 9 d following therapeutic ACT; *n* = 8 mice per group. Data represent two independent experiments; **P = 0.0074; two-tailed unpaired *t*-test. **b**, Quantification of CD90.1^+^ adoptively transferred cells in the TILs of mice 9 d following therapeutic ACT. *n* = 8 mice per group; data represent two independent experiments; ****P = 0.00004; two-tailed unpaired *t*-test. **c**, PCA plot depicting TIL- or tdLN-derived CD90.1^+^ cells from either IL-2 or IL-7/IL-15 culture. **d**, Volcano plot highlighting differentially expressed genes between tdLN and TIL samples. *n* = 4 samples per group. *P* values were determined by the Wald test and adjusted for multiple tests using the Benjamini–Hochberg algorithm. Significant genes were considered as those with an adjusted *P* value < 0.05 and an absolute log fold change >1. **e**, Representative flow cytometry plots (left) and quantification (right) of T cell memory subsets present among CD90.1^+^ cells in the tdLNs. *n* = 4 mice per group, representative of two independent experiments. **f**, Quantification of CD90.1^+^ T_SCM_ present in tdLNs of mice receiving therapeutic ACT with either IL-2- or IL-7/IL-15-treated cells. *n* = 4 mice per group, representative of two independent experiments; **P = 0.0019; two-tailed unpaired *t*-test. **g**, Representative flow cytometry plots (left) and quantification (right) of T cell memory subsets present among CD90.1^+^ cells in TILs. *n* = 4 mice per group, representative of two independent experiments. **h**, Quantification of CD62L expression by CD90.1^+^ T cells in the tdLNs or TILs of mice following therapeutic ACT. *n* = 4 mice per group, representative of two independent experiments; ***P = 0.0001; ****P = 0.00003; two-way ANOVA with Šidák correction for multiple comparisons. All data represented as mean ± s.e.m. Source data

To further investigate the cellular programs of therapeutic CLTC_H129>Q_-specific T_SCM_-like cells in vivo, CD90.1^+^CD4^+^ T cells were sorted from tdLNs and TILs 9 d after adoptive transfer of CD4^+^ T cells expressing TCR 1 differentiated in either IL-2 or IL-7/IL-15. A significant increase in the frequency of CD90.1^+^CD4^+^ T cells was observed in both the tdLNs and TILs of mice receiving IL-7/IL-15-conditioned T cells compared to those cultured in IL-2, consistent with trends in the peripheral blood (Fig. 6a,b). Next, we performed bulk RNA sequencing (RNA-seq) to compare the transcriptomic features of cells treated with IL-2 or IL-7/IL-15 in these two tissue compartments. Principal-component analysis (PCA) revealed clustering of tdLN samples and TIL samples together, suggesting that the microenvironment promotes consistent transcriptomic features independent of cytokine treatment (Fig. 6c). To assess tissue-specific genetic signatures, we investigated differentially expressed genes between all TIL and tdLN samples regardless of cytokine treatment. Among genes differentially expressed in the tdLNs were *tcf7* and *sell*, markers of memory T cells, whereas cells from TILs expressed genes associated with effector functions, including type 1 helper T (T_H_1) cytokines (*tnf*), TCR-signaling pathway components (*fos*) and cytotoxicity genes (*prf1*, *gzmc*, *gzme* and *gzmf*) (Fig. 6d). Therefore, we decided to further investigate the memory phenotypes of adoptively transferred cells in the tdLNs and TILs by flow cytometry. Consistent with our RNA-seq data, we identified memory subpopulations corresponding to effector memory (T_EM_) cells (CD44^+^CD62L^−^), central memory T (T_CM_) cells (CD44^+^CD62L^+^) and T_SCM_ cells (CD44^−^CD62L^+^) within the tdLNs (Fig. 6e). Notably, we identified a population of CD90.1^+^ T_SCM_ cells within the lymph nodes of mice receiving cells treated with IL-7/IL-15 that was almost entirely absent in mice receiving IL-2-treated cells (Fig. 6f). In both groups, CD90.1^+^ cells within the tumor were almost entirely of the T_EM_ phenotype, with little to no apparent expression of CD62L (Fig. 6g,h). These data suggest that adoptively transferred in vitro-generated T_SCM_ cells are selectively maintained as a reservoir in the tdLNs, where they give rise to more differentiated subsets capable of trafficking to the TME.

### ACT with CLTC_H129>Q_-specific CD4^+^ T cells alters host CD8^+^ T cell phenotypes

**Fig. 7:**
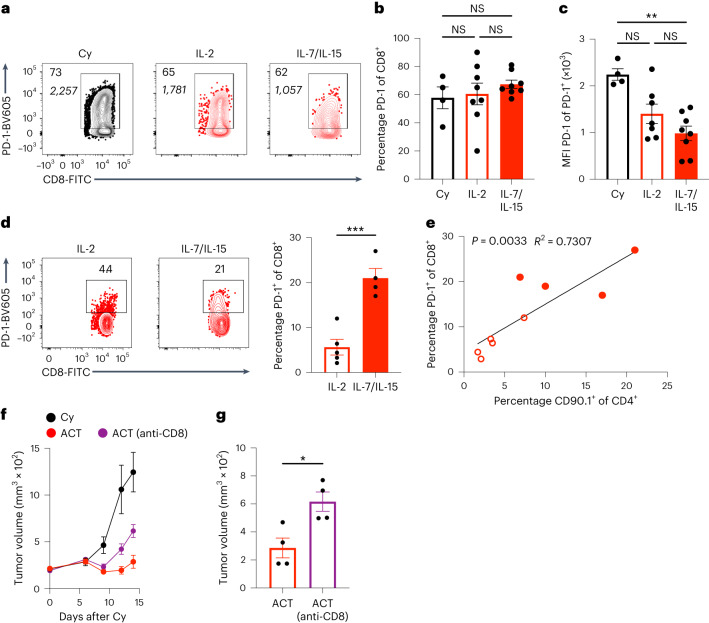
Therapeutic ACT with T_SCM_-like CLTC_H129>Q_-specific CD4^+^ T cells requires host CD8^+^ T cells. **a**, Representative flow cytometry plots indicating PD-1 expression by CD8^+^ T cells from tumors collected 9 d after treatment with percentage (top) and MFI (bottom) indicated. **b**, Quantification of frequency of PD-1^+^CD8^+^ T cells from tumors. *n* = 4 mice for Cy treatment and *n* = 8 mice for IL-2 and IL-7/IL-15 ACT-treated groups. Data represent two independent experiments. NS, *P* > 0.05; two-way ANOVA. **c**, Quantification of PD-1 MFI of PD-1^+^CD8^+^ T cells from tumors. *n* = 4 mice for Cy treatment and *n* = 8 mice for IL-2 and IL-7/IL-15 ACT-treated group; data represent two independent experiments; **P = 0.0042; two-way ANOVA with Šidák correction for multiple comparisons. **d**, Representative flow cytometry plots (left) and quantification (right) of PD-1^+^CD8^+^ T cells present in the tdLNs of mice receiving therapeutic ACT. *n* = 5 mice for IL-2 and *n* = 4 mice for IL-7/IL-15 groups, representative of two independent experiments. ***P = 0.0008; two-tailed unpaired *t*-test. **e**, Pearson correlation of the frequency of adoptively transferred CD90.1^+^CD4^+^ T cells versus the frequency of PD-1^+^CD8^+^ T cells in the tdLNs. *n* = 5 mice for IL-2 and 4 mice for IL-7/IL-15 groups, representative of two independent experiments. *R*^2^ and two-tailed *P* value determined by Pearson correlation. **f**, Tumor growth curves of mice treated with cyclophosphamide ± 3 × 10^6^ TCR 1-expressing cells treated with IL-7/IL-15 with or without CD8 depletion. *n* = 4 mice per group. Data are representative of two independent experiments. **g**, Tumor volumes from treated mice from **f** 14 d following treatment with or without CD8 depletion. *P = 0.0014 two-tailed unpaired *t*-test. All data represent mean ± s.e.m. Source data

To determine whether therapeutically transferred CLTC_H129>Q_-specific CD4^+^ T cells are modulating CD8^+^ T cell immunity, we investigated the phenotypes of CD8^+^ T cells in the TILs and tdLNs of mice receiving therapeutic ACT. While Cy is lymphodepleting, other studies using a similar dose of the drug demonstrate that full lymphocyte recovery is apparent between 5–10 d following treatment^23,24^, suggesting that endogenous host lymphocytes are available for interactions with adoptively transferred CD4^+^ T cells. In the TME, there were no significant differences in the percentage of CD8^+^ T cells expressing PD-1 (Fig. 7a,b). Given that higher levels of PD-1 expression correlate with terminally exhausted cell states^25^, we also investigated the level of PD-1 expression by analyzing the MFI of PD-1^+^CD8^+^ T cells. We found that despite the similar absolute frequency of PD-1^+^ cells, CD8^+^ TILs from mice treated with T_SCM_-like CD4^+^ T cells cultured in IL-7/IL-15 expressed significantly lower levels of PD-1 compared to CD8^+^ T cells from mice treated with Cy alone, suggesting that these CD8^+^ T cells may be less terminally exhausted (Fig. 7c).

Given that CD4^+^ T cell help for the priming of antigen-specific CD8^+^ T cells is believed to occur in the local lymph nodes, we also investigated CD8^+^ T cell phenotypes in the tdLNs of mice receiving therapy. Specifically, we again looked at PD-1 expression, which is known to correlate with stem-like, tumor-specific T cells in the tdLNs^26^. Notably, we found a significant increase in the frequency of PD-1^+^CD8^+^ T cells in the tdLNs of mice receiving adoptively transferred cells cultured in IL-7/IL-15 as compared to mice receiving cells cultured in IL-2 (Fig. 7d). Furthermore, there was a significant positive correlation between the frequency of CD90.1^+^ adoptively transferred CD4^+^ T cells and the frequency of PD-1^+^CD8^+^ T cells in lymph nodes from both IL-2 and IL-7/IL-15-treated mice (Fig. 7e). Overall, these data suggest that adoptively transferred T_SCM_-like CD4^+^ T cells recognizing CLTC_H129>Q_ are actively involved in the priming of tumor-specific CD8^+^ T cells in the tdLNs.

To confirm the role of host CD8^+^ T cells in therapeutic ACT experiments, we treated mice with IL-7/IL-15-treated CD4^+^ T cells expressing TCR 1 with or without concurrent antibody-mediated depletion of CD8^+^ T cells. While initially, following treatment, CD8-depleted mice exhibited delayed tumor growth compared to mice treated with Cy alone, ultimately CD8-depleted mice had significantly increased tumor burden relative to treated mice without depletion (Fig. 7f,g). These results confirm that endogenous host CD8^+^ T cells are required for therapeutic efficacy of ACT with CLTC_H129Q_-specific CD4^+^ T cells.

## Discussion

In this study we have analyzed an oligoclonal CD4^+^ T cell response to a naturally arising tumor NeoAg at the level of TCR usage and functionality. Although there has been a greater emphasis on CD8^+^ T cell responses in this context, perhaps due to the fact that they can directly recognize most tumors and the comparative ease in identifying the target NeoAgs presented by MHC-I versus MHC-II, the fact that CD4^+^ T cells are crucial for the priming and regulation of CD8^+^ T cells suggests that a deeper understanding of their response to cancer could significantly improve existing immunotherapies, including ACT. In this study, we investigated how differences in TCR characteristics and T cell functional states impact the efficacy of ACT with NeoAg-specific CD4^+^ T cells.

Cloning multiple CLTC_H129>Q_-specific TCRs from SCC VII-immune mice allowed us to investigate how TCR-binding kinetics may impact T cell-intrinsic and extrinsic factors contributing to the antitumor immune response. We demonstrate that while CLTC_H129>Q_-specific TCR 3 has a comparatively lower avidity for peptide–MHC complexes than TCR 1, CD4^+^ T cells expressing either TCR are similarly able to transduce TCR signaling, proliferate in vivo, contribute to CD8^+^ T cell- and CD40L-dependent primary tumor immunity and provide therapeutic tumor control. These data suggest that across the range investigated in this study, TCR avidity does not significantly affect cell-intrinsic TCR signaling or cell-extrinsic interactions providing CD40L stimulation to accessory antigen-presenting cells (APCs), as is likely required for effective licensing of dendritic cells and subsequent priming of CD8^+^ T cells in our model. These results are consistent with studies in chronic infection models suggesting that tetramer-binding avidity may not correlate with CD4^+^ T cell function or fate. Specifically, a study of lymphocytic choriomeningitis virus infection in mice found that tetramer-negative low affinity CD4^+^ T cells exist at a similar frequency to tetramer-positive cells and contribute inflammatory cytokines during the effector phase^27^. Other studies suggest that the off-rate (*K*^off^) of TCR interactions with peptide–MHC complexes may play a role in the commitment of individual clonotypes to distinct T_H_ cell and memory lineages, whereas TCR avidity as measured by tetramer-binding studies alone did not correlate with either^28,29^. Notably, recent studies of MHC-I-restricted TCRs recognizing tumor antigens with a similar range of tetramer-binding capacity to those in our study did observe a correlation between TCR avidity and T cell functions in vitro and in vivo, suggesting that the possibility that CD4^+^ and CD8^+^ T cell subsets and their respective functions have different TCR avidity requirements^18,30^. One limitation of our study is the requirement for T cell activation before TCR expression by retroviral transduction; we were therefore unable to determine whether TCR avidity differentially regulates T cell activation and priming of naive T cells during primary tumor immunity. Furthermore, the TCRs identified in this study were isolated from polyclonal CD4^+^ T cells following tumor rejection, likely reflecting a memory population; TCRs collected from the effector phase during tumor growth may have more diverse functional properties.

It is notable that SCC VII tumor cells do not express MHC-II, even after treatment with IFN-γ. While transcriptomic signatures corresponding to cytotoxic CD4^+^ T cells have been identified in patients with melanoma and bladder cancer^31,32^ and direct tumor recognition by CD4^+^ T cells has been observed^33,34^, studies also suggest that only a small subset of melanomas harbor any MHC-II^+^ cells^35^. Even among MHC-II^+^ tumor cells, endogenous antigens are selectively presented by MHC-II, which may limit direct recognition of critical tumor NeoAg^36^. A recent study from Rosenberg and colleagues demonstrated that among 20 confirmed NeoAg-specific TCRs isolated from CD4^+^ TILs found in human tumors, none was able to directly recognize autologous tumor cells^37^. Despite the inability of SCC VII cells to express MHC-II, cells expressing TCR 1 within the TME were enriched for transcripts associated with cytotoxicity (*prf1*, *gzmc*, *gzme* and *gzmf*), suggesting that this transcriptomic signature may be broadly associated with local CD4^+^ effector T cell differentiation even in cases where such cells cannot directly engage tumor cells. Overall, MHC-II^−^ solid tumor models such as SCC VII may more closely model human disease and are an important alternative to murine cancer cells with inducible or constitutive MHC-II expression.

While SCC VII cells do not express MHC-II, ACT with T_SCM_-like TCR-engineered CD4^+^ T cells generates effective therapeutic immunity in the context of established solid tumors that is dependent on CD8^+^ T cells and likely mediated by APC activation (‘licensing’) via CD40/CD40L interactions^38^. Several studies now implicate migratory cDC1 as the recipient of CD40L stimulation from CD4^+^ T cells in humans and mice^20,39,40^, suggesting that these are the relevant APC in our study. Consistent with our hypothesis of improved CD8^+^ T cell priming, we observed a significant increase in the frequency of PD-1^+^ CD8^+^ T cells in the tdLNs during effective therapeutic ACT. In addition, CD8^+^ TILs in mice treated with T_SCM_-like CD4^+^ T cells expressed lower levels of PD-1 in the TME, consistent with previously published results suggesting that CD8^+^ T cells primed in the absence of help express higher levels of co-inhibitory receptors^41^. While our results implicate a role for T cell help in the tdLNs, we cannot rule out additional cooperative functions locally within the TME between newly primed CD8^+^ T cells and NeoAg-specific CD4^+^ T cells, such as CD4^+^ T cell-derived IL-2 and IFN-γ, which serve to support CD8^+^ T cell survival in and recruitment to tumors, respectively^42^. Indeed, our data suggest that adoptively transferred CD4^+^ T cells are also capable of recognizing antigen in the TME, where they express a transcriptomic signature associated with effector function and differentiation. In addition, given that CD8^+^ T cell depletion does not completely abrogate antitumor efficacy in our therapeutic models, it is likely that additional mechanisms beyond APC licensing, such as local effector cytokine secretion, are required.

To improve the persistence of adoptively transferred CD4^+^ T cells, we administered Cy as a lymphodepletion regimen before ACT. In addition to depleting inhibitory lymphocytes and increasing homeostatic cytokine levels, lymphodepleting regimens have been reported to promote the release of innate immune ligands such as lipopolysaccharide (LPS) from gut microbiota, which potentiates host dendritic cell activation^43^. Notably, C3H/HeJ mice have a missense mutation in TLR4, which likely reduces the impact of this signaling axis following lymphodepletion and may therefore underestimate the efficacy of Cy in our model.

The superior efficacy of IL-7/IL-15-treated CD4^+^ T cells merits further investigation. While previous studies have demonstrated an enhanced capacity for expansion and therapeutic efficacy of CD4^+^ T cells primed in the presence of IL-7 or expressing a constitutively active mutant of STAT5 (refs. ^44,45^), our study extends these findings by demonstrating that IL-7/IL-15-treated CD4^+^ T cells adopt a unique surface phenotype in vitro associated with T_SCM_-like cells, which has mainly described in the context of tumor-specific CD8^+^ T cells. Other cytokines and treatments, such as IL-21, IL-9 and Wnt pathway inhibitors, have been described as inducers of T_SCM_-like CD8^+^ T cells and may similarly promote this phenotype in CD4^+^ T cells^21,46,47^. While IL-7 and IL-15 were used at a single concentration in our study, it is possible that altering the concentration of these cytokines may improve the yield of T_SCM_-like cells in this context. Our data demonstrate that following adoptive transfer, TCR-engineered T_SCM_-like cells generated in culture with IL-7/IL-15 maintain a reservoir in the tdLNs accounting for nearly a quarter of all CD90.1^+^ cells, whereas tumor-infiltrating cells express a differentiated effector memory phenotype. Several recent studies have highlighted the tdLNs as a crucial site for the maintenance of CD8^+^ tumor-specific T cells expressing memory-associated features, which seem critical for responses to ICB^26,48,49^. Our results suggest that these tdLN-resident memory cell populations may be effectively installed via ACT in patients lacking a sufficient tumor-specific memory cell reservoir and highlight the potential for combination therapies with ICB.

ACT with NeoAg-specific CD4^+^ T cells may have important advantages over ACT with CD8^+^ T cells. First, studies suggest that in both murine models and human cancers, NeoAg-specific CD4^+^ T cells may be more abundant than NeoAg-specific CD8^+^ T cells. In preclinical and clinical studies of personalized cancer vaccines, epitopes selected for binding to MHC-I perhaps surprisingly predominantly gave rise to CD4^+^ T cell responses^6,50^. In our own functional NeoAg screening approach applied to SCC VII, which does not leverage bioinformatic MHC binding predictions, we identified four MHC-II-restricted NeoAg and only one MHC-I-restricted NeoAg following vaccination with irradiated tumor cells^16^. This suggests that there may be a relative abundance of MHC-II-restricted TCRs available for immunotherapy, including previously identified TCRs specific for shared oncogenic driver mutations such as BRAF V600E^11^, KRAS G12V^36,51^ and G12D^4^ and IDH1 R132H^52^. In addition, by operating independently of direct tumor recognition, such as by marshaling a polyclonal CD8^+^ T cell response, ACT with CD4^+^ T cells may circumvent immune escape mechanisms associated with monoclonal CD8^+^ T cell ACT^53,54^. Overall, our study demonstrates the efficacy of ACT with NeoAg-specific CD4^+^ T cells in a physiologically relevant tumor model and brings new insights to the use of similar approaches for adoptive immunotherapy of human cancer to empower more diverse, potent and durable antitumor immune responses.

## Methods

### Animals

Female C3H/HeJ mice (The Jackson Laboratory) were used in these experiments. Animals were 8–12 weeks of age and maintained/bred in The La Jolla Institute for Immunology vivarium under specific-pathogen-free conditions in accordance with guidelines of the Association for Assessment and Accreditation of Laboratory Animal Care International and animal studies were approved by The La Jolla Institute for Immunology Institutional Animal Care and Use Committee.

### Cell culture

The squamous cell carcinoma VII San Francisco line (SCC VII) spontaneously arose from the abdominal wall of a C3H mouse in the laboratory of Herman Suit (Harvard University) and was adapted for partial in vitro growth by K.K. Fu and K.N. Lam (University of California). SCC VII was maintained for a maximum of three passages in vitro in RPMI 1640 medium containing 10% fetal bovine serum (FBS) and penicillin–streptomycin (100 U ml^−1^ each, Gibco). To regenerate SCC VII P_0_ cells, C3H/HeJ mice were subcutaneously (s.c.) injected with 5 × 10^5^ cells in 1× HBSS and tumors were collected 14 d after inoculation. Tumor tissue was dissociated with a mouse Tumor Dissociation kit (Miltenyi Biotec) followed by passage through a 70-µm cell strainer (Fisher Scientific) and homogenized cells were re-seeded in vitro. For generation of SCC VII expressing luciferase and copepod-derived GFP (SCC VII-Luc/GFP), cells were transduced with the BVLIV713VA-1 HIV lentiviral vector (System Biosciences) under 10 µg ml^−1^ puromycin selection and further purified using GFP + FACS-sorting. The 58α^−^β^−^ hybridoma cell line was cultured in RPMI medium supplemented with l-glutamine and HEPES (10 mM, Gibco), 10% FBS, 1 mM sodium pyruvate (Gibco), 1× MEM Non-Essential Amino Acids (Gibco) and penicillin–streptomycin (100 U ml^−1^ each, Gibco).

### Whole-cell vaccination and tumor challenge

Whole tumor cell vaccination experiments were conducted via s.c. injection of 10 × 10^6^ 50 Gy-irradiated SCC VII cells in 1× HBSS with 50 µg polyI:C (Thermo Fisher). Immunized mice were challenged 14 d later by s.c. injection of 5 × 10^5^ live SCC VII cells.

### Preparation of single-cell suspensions

Spleens, inguinal lymph nodes and tumors were surgically removed at experiment end points. Spleens were dissociated manually and cell suspensions were passed through a 70-µm strainer. Before use as APCs in in vitro assays, red blood cells were lysed with ACK lysis buffer (Thermo Fisher). Inguinal lymph nodes and subcutaneous tumors were minced into small (<2 mm) pieces with dissection scissors. Tissue fragments were enzymatically dissociated in 20 µg ml^−1^ Liberase (Roche) and 20 µg ml^−1^ DNase I (Roche) at 37 °C for 30 min. Single cells were passed through a 70-µm strainer.

### TCR sequencing

Single tetramer-positive CD4^+^ T cells from the spleens of SCC VII-immune mice 14 d after challenge were sorted into 96-well plates using an FACS Fusion (BD). Multiplexed PCR amplification of the TCR α and α variable regions was performed as previously described^17^. cDNA libraries were sequenced by Sanger sequencing (ETON). Full-length TCR sequences were reconstructed from cDNA fragments using the IMGT database to identify corresponding V and J gene usage^55^. IMGT nomenclature for TCR V and J genes is used throughout the manuscript for consistency.

### TCR cloning and expression

TCR nucleotide sequences were synthesized and cloned into MSGV1 retroviral expression backbones using a BioXP (Codex DNA). TCR β and α-chains were separated by a P2A ribosomal skipping element. For in vivo studies, constructs were synthesized encoding the TCR β and α-chains as described above, followed by an additional P2A sequence and the coding sequence of CD90.1. Spleens from naive C3H/Hej mice were dissociated manually and cell suspensions passed through a 70-µm filter. CD4^+^ T cells were isolated by magnetic negative selection (StemCell). CD4^+^ T cells were stimulated with anti-CD3/CD28 Dynabeads (Gibco) for 24 h. TCR retroviral supernatants were generated by co-transfection of Platinum-Eco cells with the TCR containing retroviral vectors and pcL-ECo plasmid. Retroviral supernatants were collected at 48 and 72 h after transfection and either used fresh or frozen at −80 °C. Transductions were performed on RetroNectin-coated plates (Takara) as previously described. Murine T cells were maintained in RPMI 1640 supplemented with 10% FBS, 50 µM β-mercaptoethanol, 1× penicillin–streptomycin and HEPES supplemented with 100 IU ml^−1^ human IL-2 (Roche) or 5 ng ml^−1^ human IL-7 and IL-15 (StemCell) in the presence of anti-CD3/CD28 Dynabeads (Thermo Fisher) for expansion and used for experiments between 10–14 d after isolation. Dynabeads were magnetically removed before use in in vitro assays or adoptive transfer experiments.

### Flow cytometry

Splenocytes and transduced T cells were stained with the indicated concentration I-A^K^(VALVTDNAVYQWSME)-PE tetramer for 1 h at 37 degrees. Before surface staining, Fc receptors were blocked with anti-CD16/32 TruStain FcX (BioLegend) for 15 min on ice. Cells were then washed and stained for surface antigens on ice for 15–30 min. Dead cells were excluded with either 4,6-diamidino-2-phenylindole (DAPI), LIVE/DEAD Fixable Yellow (Thermo Fisher) or Fixable Viability Dye eFluor 780 (Thermo Fisher). Fluorescently conjugated antibodies specific for the following murine antigens were used in this study: CD45 (30-F11, 103131, BioLegend), CD4 (RM4-5, 100509, BioLegend), Thy1-1 (OX-7, 202523, BioLegend), TRBV8.3 (1B3.3, 553663, BD Biosciences), PD-1 (29F.1A12, 135219, BioLegend), CD69 (H1.2F3, 104513, BioLegend), TRBV14 (J9.19, 553258, BD Biosciences), TRBV4 (KT4, 553365, BD Biosciences), TCRb (H57-597, 20-5961, Tonbo), pERK1/2 (4B11B69, 675507, BioLegend), I-A/I-E (M5/114.15.2, 17-5321-82, eBiosciences), B220 (RA3-6B2, 25-0452-82, Invitrogen), CD3 (17A2, 100217, BioLegend), CD62L (MEL-14, 20-0621-U025, Tonbo), CD44 (IM7, 561860, BD Biosciences), Ly-6A/E (F13-161.7, 108123, BioLegend), CD8 (53-6.7, 35-0081-U025, Tonbo) and CD40L (MR1, 106505, BioLegend). All surface-staining antibodies listed were used at a 1:200 dilution. Anti-pERK1/2 was used at a 1:50 dilution for intracellular staining. Data were collected on a BD FACS Celesta or BD LSR-II using BD FACSDiva software and analyzed using FlowJo.

### In vitro T cell functional assays

For splenocyte co-culture assays, 2 × 10^4^ transduced T cells were co-cultured with 2 × 10^5^ splenocytes pulsed overnight with indicated concentrations of CLTC_H192>Q_ (SLNTVALVTDNAVYQWSMEG) or wild-type CLTC (SLNTVALVTDNAVYHWSMEG) peptide in 96-well U-bottom plates. Supernatants were collected after 18–24 h and IFN-γ was measured by ELISA (BD Bioscience). For measuring levels of proximal TCR signaling, 5 × 10^4^ transduced T cells were co-cultured with 1 × 10^5^ splenocytes pulsed overnight with 1 µg ml^−1^ CLTC_H129>Q_ peptide in 96-well U-bottom plates. Cells were centrifuged for 10 s at 400*g* to initiate contact between T cells and APCs. After incubation for 5 min at 37 °C, the reaction was stopped on ice for 30 s. The plate was then centrifuged at 311*g* for 2 min, supernatants were discarded and wells were vortexed to resuspend cells in remaining volume. Cells were immediately fixed with ice-cold 4% paraformaldehyde for 15 min on ice, followed by two washes with FACS buffer. Cells were then permeabilized with ice-cold 90% methanol for 15 min, followed by an additional two washes with FACS buffer. Cells were then stained intracellularly for phosphorylated ERK1/2 for 30 min at room temperature, before washing twice with FACS buffer and analyzing levels of phosphorylated ERK1/2 by flow cytometry.

### Adoptive transfers and in vivo treatments

Naive or tumor-bearing C3H/HeJ mice were injected i.v. via the tail vein with the indicated number of CD4^+^ T cells in 200 µl 1× HBSS at the indicated time points. Where indicated, CD4^+^ T cells were first labeled with CTV (Thermo Fisher) according to manufacturer’s instructions. Depletion of CD8^+^ T cells was achieved by intraperitoneal (i.p.) injection of 200 µg anti-CD8 (116-13.1, BE0118, BioXCELL) or IgG2a (C1.18.4, BE0085, BioXCELL) isotype control at D4 and D0 relative to tumor cell injection for primary tumor immunity experiments. To deplete CD8^+^ T cells during therapeutic experiments, 200 µg anti-CD8 was injected i.p. immediately following adoptive transfer of CD4^+^ T cells and every 7 d for the duration of the experiment. CD40L was blocked in vivo by i.p. administration of 200 µg anti-CD40L (MR1, BE0017-1, BioXCELL) compared to Armenian hamster IgG (PIP, BE0260, BioXCELL) isotype control on D0 and D2 for primary tumor immunity experiments. Where indicated, tumor-bearing mice were treated with 150 mg kg^−1^ cyclophosphamide monohydrate (Sigma) dissolved in 1× PBS i.p. 1 d before T cell transfer. Tumor volume was calculated by the equation *V* = (*l* × *w*^2^) / 2 where *l* and *w* correspond to the longer and shorter perpendicular diameters respectively. For therapeutic experiments, mice were treated once tumor volumes reached 100–250 mm^3^. Mice were randomized before initiating treatment.

### RNA sequencing and bioinformatic analyses

RNA paired-end sequencing reads were obtained using Illumina’s NovaSeq 6000 system. FastQC (v.0.11.9) and Trimmomatic (v.0.32) were used to run quality control and trim low-quality control reads. The paired ends that passed Illumina filters were filtered for reads aligning to tRNA, rRNA, adaptor sequences and spike-in controls. The reads were then aligned to the GRCm38 reference genome and Gencode v.M9 annotations using STAR (v.2.6.1c)^56^. DUST scores were calculated with PRINSEQ Lite (v.0.20.3)^57^ and low-complexity reads (DUST > 4) were removed from the BAM files. The alignment results were parsed via SAMtools^58^ to generate SAM files. Read counts to each genomic feature were obtained with the featureCounts program (v.1.6.5)^59^. After removing absent features (zero counts in all samples), the raw counts were then imported to R/Bioconductor package DESeq2 (ref. ^60^) to identify differentially expressed genes among samples. *P* values for differential expression were calculated using the Wald test for differences between the base means of two conditions. These *P* values were then adjusted for multiple test correction using the Benjamini–Hochberg algorithm^61^ to control the false discovery rate. We considered genes differentially expressed between two groups of samples when the DESeq2 analysis resulted in an adjusted *P* value of <0.05 and the absolute value of the log fold change in gene expression was >1. Variance stabilizing transformation (DESeq2 v.1.24.0) was applied to the read counts for all samples. As there were samples from multiple mapping runs, adjustment of batch effect with outcome of interest as disease state was performed using ComBat (sva v.3.32.1).

### Statistics

Statistical analyses were performed with Prism 9 (GraphPad Software). Statistical tests and significance are indicated in the figure legends.

### Study approval

All animal studies were approved by The La Jolla Institute for Immunology Institutional Animal Care and Use Committee Animal Protocol AP00001026.

### Reporting summary

Further information on research design is available in the Nature Portfolio Reporting Summary linked to this article.

## Online content

Any methods, additional references, Nature Portfolio reporting summaries, source data, extended data, supplementary information, acknowledgements, peer review information; details of author contributions and competing interests; and statements of data and code availability are available at 10.1038/s41590-023-01543-9.

## Supplementary information

Reporting Summary

## Data Availability

Bulk RNA-seq data have been uploaded to the NCBI Gene Expression Omnibus and are accessible under accession no. GSE229221. The mouse reference genome GRCm38 is accessible through GenBank under accession no. GCA_000001635.2. Source data are provided with this paper.

## Source data

Source Data Fig. 1 Statistical source data.
Source Data Fig. 2 Statistical source data.
Source Data Fig. 3 Statistical source data.
Source Data Fig. 4 Statistical source data.
Source Data Fig. 5 Statistical source data.
Source Data Fig. 6 Statistical source data.
Source Data Fig. 7 Statistical source data.
Source Data Extended Data Fig. 2 Statistical source data.
Source Data Extended Data Fig. 3 Statistical source data.
Source Data Extended Data Fig. 4 Statistical source data.
Source Data Extended Data Fig. 5 Statistical source data.
