# Author Correction: Neoantigen-specific stem cell memory-like CD4^+^ T cells mediate CD8^+^ T cell-dependent immunotherapy of MHC class II-negative solid tumors

**DOI:** 10.1038/s41590-023-01591-1

**Published:** 2023-07-18

**Authors:** Spencer E. Brightman, Angelica Becker, Rukman R. Thota, Martin S. Naradikian, Leila Chihab, Karla Soria Zavala, Ashmitaa Logandha Ramamoorthy Premlal, Ryan Q. Griswold, Joseph S. Dolina, Ezra E. W. Cohen, Aaron M. Miller, Bjoern Peters, Stephen P. Schoenberger

**Affiliations:** 1 Division of Developmental Immunology, La Jolla Institute for Immunology, La Jolla, CA USA; 2 Biomedical Sciences Program, School of Medicine, University of California San Diego, La Jolla, CA USA; 3 Division of Vaccine Discovery, La Jolla Institute for Immunology, La Jolla, CA USA; 4 Division of Hematology and Oncology, University of California San Diego Moores Cancer Center, UCSD, La Jolla, CA USA; 5 Department of Medicine, University of California San Diego, La Jolla, CA USA

**Keywords:** Immunology, Diseases

Correction to: *Nature Immunology* 10.1038/s41590-023-01543-9. Published online 3 July 2023.

In the version of this article initially published, Ashmitaa Logandha Ramamoorthy Premlal (Division of Vaccine Discovery, La Jolla Institute for Immunology, La Jolla, CA, USA), who contributed to the bioinformatic analysis of TCR genes, was missing from the author list. In Fig. 4e, the red dotted key reading “0.3” appeared originally as “3”. In the Competing interests statement, Bjoern Peters was not listed as an inventor on patents related to tumor-specific TCRs. The HTML and PDF versions of the article are now updated with the changes.

