# Supplementary material for: Neoantigen-specific stem cell memory-like CD4+ T cells mediate CD8+ T cell-dependent immunotherapy of MHC class II-negative solid tumors
Source: Nat Immunol. 2023 Jul 3;24(8):1345–57. doi: 10.1038/s41590-023-01543-9 (PMC10382322; doi:10.1038/s41590-023-01543-9)
Supplement: Supplementary file 1 — Reporting Summary [file 41590_2023_1543_MOESM1_ESM.pdf]

Corresponding author(s): Stephen P. Schoenberger
Last updated by author(s): 5/16/2023

Reporting Summary

Nature Portfolio wishes to improve the reproducibility of the work that we publish. This form provides structure for consistency and transparency in reporting. For further information on Nature Portfolio policies, see our Editorial Policies and the Editorial Policy Checklist.

Statistics

For all statistical analyses, confirm that the following items are present in the figure legend, table legend, main text, or Methods section.

n/a	Confirmed	
☐	☒	The exact sample size (*n*) for each experimental group/condition, given as a discrete number and unit of measurement
☐	☒	A statement on whether measurements were taken from distinct samples or whether the same sample was measured repeatedly
☐	☒	The statistical test(s) used AND whether they are one- or two-sided<br>*Only common tests should be described solely by name; describe more complex techniques in the Methods section.*
☐	☒	A description of all covariates tested
☐	☒	A description of any assumptions or corrections, such as tests of normality and adjustment for multiple comparisons
☐	☒	A full description of the statistical parameters including central tendency (e.g. means) or other basic estimates (e.g. regression coefficient) AND variation (e.g. standard deviation) or associated estimates of uncertainty (e.g. confidence intervals)
☐	☒	For null hypothesis testing, the test statistic (e.g. *F*, *t*, *r*) with confidence intervals, effect sizes, degrees of freedom and *P* value noted<br>*Give P values as exact values whenever suitable.*
☒	☐	For Bayesian analysis, information on the choice of priors and Markov chain Monte Carlo settings
☒	☐	For hierarchical and complex designs, identification of the appropriate level for tests and full reporting of outcomes
☐	☒	Estimates of effect sizes (e.g. Cohen's *d*, Pearson's *r*), indicating how they were calculated

Our web collection on statistics for biologists contains articles on many of the points above.

Software and code

Policy information about availability of computer code

Data collection	BD FACSDiva was used to collect flow cytometry data
Data analysis	Flow cytometry data were analyzed using FlowJo v10.8.2. Data analysis, visualization, and statistical analyses were performed with Prism 9. For RNA-seq data, the following analysis packages were used: FastQC (v0.11.9), Trimmomatic (v.0.32), STAR (v2.6.1c) , PRINSEQ Lite (v 0.20.3), SAMtools (v1.17), featureCounts (v1.6.5), DESeq2 (1.24.0), ComBat (3.32.1)

For manuscripts utilizing custom algorithms or software that are central to the research but not yet described in published literature, software must be made available to editors and reviewers. We strongly encourage code deposition in a community repository (e.g. GitHub). See the Nature Portfolio guidelines for submitting code & software for further information.

Data

Policy information about availability of data

All manuscripts must include a data availability statement. This statement should provide the following information, where applicable:

- Accession codes, unique identifiers, or web links for publicly available datasets
- A description of any restrictions on data availability
- For clinical datasets or third party data, please ensure that the statement adheres to our policy

Data Availability Statement: Bulk RNA-seq data has been uploaded to NCBI GEO and is accessible under accession number GSE229221. Source data are provided with this article.

Human research participants

Policy information about studies involving human research participants and Sex and Gender in Research.

Reporting on sex and gender	N/A
Population characteristics	N/A
Recruitment	N/A
Ethics oversight	N/A

Note that full information on the approval of the study protocol must also be provided in the manuscript.

Field-specific reporting

Please select the one below that is the best fit for your research. If you are not sure, read the appropriate sections before making your selection.

☒ Life sciences ☐ Behavioural & social sciences ☐ Ecological, evolutionary & environmental sciences

For a reference copy of the document with all sections, see nature.com/documents/nr-reporting-summary-flat.pdf

Life sciences study design

All studies must disclose on these points even when the disclosure is negative.

Sample size	Sample sizes for animal experiments were chosen in line with published results for similar experiments.
Data exclusions	No data were excluded from this study.
Replication	All experiments were replicated at least once with number of replicate experiments indicated in the figure legends.
Randomization	For therapeutic animal experiments, mice were randomized prior to treatment initiation to account for variation in tumor size. For prophylactic experiments where treatments were administered prior to tumor initiation, animals of the same age were used and separate cages received separate treatments.
Blinding	Blinding was not possible for in vivo tumor studies as tumor measurements were only taken by one researcher. For all other experiments blinding was not necessary.

Reporting for specific materials, systems and methods

We require information from authors about some types of materials, experimental systems and methods used in many studies. Here, indicate whether each material, system or method listed is relevant to your study. If you are not sure if a list item applies to your research, read the appropriate section before selecting a response.

Materials & experimental systems

n/a	Involved in the study	
☐	☒	Antibodies
☐	☒	Eukaryotic cell lines
☒	☐	Palaeontology and archaeology
☐	☒	Animals and other organisms
☒	☐	Clinical data
☒	☐	Dual use research of concern

Methods

n/a	Involved in the study	
☒	☐	ChIP-seq
☐	☒	Flow cytometry
☒	☐	MRI-based neuroimaging

Antibodies

Antibodies used	Fluorescently conjugated antibodies specific for the following murine antigens were used in this study: CD45 (30-F11, 103131, Biolegend), CD4 (RM4-5, 100509, Biolegend), Thy1-1 (OX-7, 202523, Biolegend), TRBV8.3 (1B3.3, 553663, BD Biosciences), PD-1 (29F.1A12, 135219, Biolegend), CD69 (H1.2F3, 104513, Biolegend), TRBV14 (J9.19, 553258, BD Biosciences), TRBV4 (KT4, 553365, BD Biosciences), TCRb (H57-597, 20-5961, Tonbo), pERK1/2 (4B11B69, 675507, Biolegend), I-A/I-E (M5/114.15.2, 17-5321-82, eBiosciences), B220 (RA3-6B2, 25-0452-82, Invitrogen), CD3 (17A2, 100217, Biolegend), CD62L (MEL-14, 20-0621-U025, Tonbo),

	CD44 (IM7, 561860, BD Biosciences), Ly-6A/E (F13-161.7, 108123, Biolegend), CD8 (53-6.7, 35-0081-U025, Tonbo), CD40L (MR1, 106505, Biolegend). All surface staining antibodies listed were used at a 1:200 dilution. Anti-pERK1/2 antibody was used at a 1:50 dilution for intracellular staining.<br><br>The following antibodies were used for in vivo depletions/blockades: anti-CD8 (116-13.1, BE0118, BioXCELL), IgG2a (C1.18.4, BE0085, BioXCELL), anti-CD40L (MR1, BE0017-1, BioXCELL), Armenian hamster IgG (PIP, BE0260, BioXCELL)
Validation	All antibodies were validated for use in mice and for the indicated applications on the manufacturers websites (flow cytometry, in vivo depletion for 116-13.1, in vivo blocking for MR1.)

Eukaryotic cell lines

Policy information about cell lines and Sex and Gender in Research

Cell line source(s)	SCC VII tumor cell lines were a gift from Michael Beckett (mbeckett@uchicago.edu) and originally derived from a spontaneous murine abdominal wall tumor in the lab of Dr. Herman Suit at Harvard University. 58a-b- were a gift from Bernard Malissen (bernardm@ciml.univ-mrs.fr
Authentication	Cell lines were not authenticated
Mycoplasma contamination	All cell lines tested negative for mycoplasma
Commonly misidentified lines (See ICLAC register)	No commonly misidentified cell lines were used.

Animals and other research organisms

Policy information about studies involving animals; ARRIVE guidelines recommended for reporting animal research, and Sex and Gender in Research

Laboratory animals	8-12 week old female C3H/Hej mice were used in these studies
Wild animals	This study did not involve wild animals
Reporting on sex	Female mice were used because the tumor cell line (SCC VII) was derived from a female mouse
Field-collected samples	This study did not involve field collected samples
Ethics oversight	The animal study protocol was approved by the La Jolla Institute for Immunology Institutional Animal Care and Use Committee (IACUC)

Note that full information on the approval of the study protocol must also be provided in the manuscript.

Flow Cytometry

Plots

Confirm that:

☒ The axis labels state the marker and fluorochrome used (e.g. CD4-FITC).

☒ The axis scales are clearly visible. Include numbers along axes only for bottom left plot of group (a 'group' is an analysis of identical markers).

☒ All plots are contour plots with outliers or pseudocolor plots.

☒ A numerical value for number of cells or percentage (with statistics) is provided.

Methodology

Sample preparation	Samples were prepared as described in the materials and methods. Mouse spleens, tumors, and inguinal lymph nodes were surgically removed. Spleens were dissociated manually and cell suspensions were passed through a 70 uM strainer. Prior to use as antigen presenting cells in T cell in vitro assays, red blood cells were lysed with ACK lysis buffer. Lymph nodes and subcutaneous tumors were minced into small (<2mm) pieces with dissection scissors. Tissue fragments were enzymatically dissociated in 20 ug/mL Liberase (Roche) and 20 ug/mL DNase I (Roche) at 37 degrees C for 30 minutes. Single cells were then passed through a 70 uM strainer.
Instrument	BD LSR-II and BD FACS Celesta were used for data aquisition
Software	Flow cytometry data was collected using BD FACSDiva software and anlyzed with FlowJo v10.8.2
Cell population abundance	For tetramer sorting experiments, given the low total number of cells, no post-sort purity assessment was made and all single cells were used for TCR sequencing experiments.

Gating strategy

Where relevant, gating strategies have been included as supplemental figures. Tetramer binding CD4+ T cells from mice were pregated as live, B220-, CD3+, mTCRb+, CD8-, CD4+ cells. In adoptive transfer experiments, transferred TCR-engineered CD4 + T cells were identified as live, CD45+, CD4+, CD90.1+ cells.

☒ Tick this box to confirm that a figure exemplifying the gating strategy is provided in the Supplementary Information.
